# Characteristic Effects of Stochastic Oscillatory Forcing on Neural Firing: Analytical Theory and Comparison to Paddlefish Electroreceptor Data

**DOI:** 10.1371/journal.pcbi.1003170

**Published:** 2013-08-15

**Authors:** Christoph Bauermeister, Tilo Schwalger, David F. Russell, Alexander B. Neiman, Benjamin Lindner

**Affiliations:** 1 Max-Planck-Institute for the Physics of Complex Systems, Dresden, Germany; 2 Bernstein Center for Computational Neuroscience and Physics Department of Humboldt University, Berlin, Germany; 3 Department of Biological Sciences and Neuroscience Program, Ohio University, Athens, Ohio, United States of America; 4 Department of Physics and Astronomy and Neuroscience Program, Ohio University, Athens, Ohio, United States of America; University College London, United Kingdom

## Abstract

Stochastic signals with pronounced oscillatory components are frequently encountered in neural systems. Input currents to a neuron in the form of stochastic oscillations could be of exogenous origin, e.g. sensory input or synaptic input from a network rhythm. They shape spike firing statistics in a characteristic way, which we explore theoretically in this report. We consider a perfect integrate-and-fire neuron that is stimulated by a constant base current (to drive regular spontaneous firing), along with Gaussian narrow-band noise (a simple example of stochastic oscillations), and a broadband noise. We derive expressions for the *n*th-order interval distribution, its variance, and the serial correlation coefficients of the interspike intervals (ISIs) and confirm these analytical results by computer simulations. The theory is then applied to experimental data from electroreceptors of paddlefish, which have two distinct types of internal noisy oscillators, one forcing the other. The theory provides an analytical description of their afferent spiking statistics during spontaneous firing, and replicates a pronounced dependence of ISI serial correlation coefficients on the relative frequency of the driving oscillations, and furthermore allows extraction of certain parameters of the intrinsic oscillators embedded in these electroreceptors.

## Author Summary

We explore how a neuron responds to a special type of input signal which is oscillatory but noisy (narrow-band noise). These fluctuations could be due to sensory input, due to oscillatory activity of a surrounding network, or due to a natural stimulus. We study theoretically the effects of noisy oscillations on an idealized model neuron, which would otherwise produce as output a series of action potentials at regular intervals. Because our model is comparably simple, we can describe the effects on ISI statistics analytically with formulas that we test against computer simulations of the model. Moreover, we can compare our theoretical predictions to experimental data from electroreceptors of paddlefish - a biological example for spiking neurons that are naturally stimulated by stochastic oscillatory input. In particular, our theory provides a simple explanation of the seemingly complicated patterns of correlations between interspike intervals, that are observed for the electro-afferents in paddlefish; the theory shows also good agreement with respect to other independent spike train statistics. Our findings further the understanding of how nervous activity is shaped by oscillatory noisy signals, which can emerge in the neural networks of the brain, in the sensory periphery, and in the environment.

## Introduction

Oscillatory activity is common in neural systems. Mechanical oscillations form an important class of sensory stimuli, for instance, in hearing, but may also be generated autonomously by mechanosensory hair cells [1]. In single neurons, periodicities may occur in the form of subthreshold membrane potential oscillations [2]. Oscillations at the level of brainstem and spinal cord neural networks generate the coordinated motor patterns for breathing and locomotion. Cortical networks may cause periodicities of local field potentials [3] or electroencephalogram (EEG) or magnetoencephalogram (MEG) activity [4].

**Figure 1:**
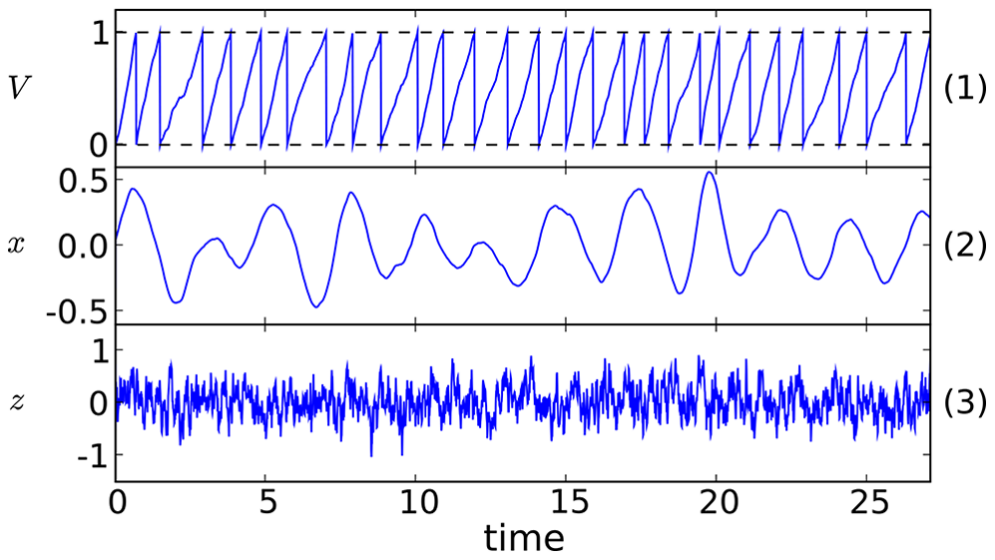
Illustration of the neuron model, showing a calculated membrane voltage trace (upper panel) that yields a spike time whenever a threshold level is reached, and sample trajectories of input narrow-band harmonic noise (middle panel) and broadband short-correlated Ornstein-Uhlenbeck (OU) noise (lower panel).

With few exceptions, e.g. motor rhythms and the precise rhythm of the electric organ discharge in weakly electric fish [5], the oscillations generated by neural systems are not coherent over long time scales, but instead show fluctuations in both phase and amplitude (see Fig. 1, middle panel, for an example). Such periodic signals with limited coherence are termed *stochastic oscillations*, and are characterized by a preferred frequency band of spectral power. An individual neuron's activity may be affected by stochastic oscillations via synaptic input to it, or from its own endogenous fluctuations. Although stochastic oscillations are frequently found in neural systems, there is generally poor understanding of how an input current of this kind affects the firing pattern of a neuron, its ability to transmit information about time-dependent stimuli, and its interaction with other cells in a neural network. This is in marked contrast to the often studied (non-stationary) problem of how a *deterministic* periodic driving affects neural activity (see e.g. [6]–[13]). The simplest yet non-trivial problem that comes up with stochastic oscillations is how they shape the *spontaneous* activity of a spiking neuron, our topic here.

For the strictly periodic (i.e. a deterministic) driving, different analytical approaches and results exist (see e.g. [8], [11], [12]). Explicit expressions for the spike statistics of neurons driven by stochastic oscillations, however, are still lacking even for simple integrate-and-fire type models (for a notable exception, see [14] for an approach to the count statistics of such models). Formulas, e.g. for the ISI statistics, are desirable for several reasons. First, the analytical approach gives us a more thorough understanding of the spike time statistics, along with opportunities to formulate falsifiable predictions from the model. Secondly, in many neurons, a stochastic oscillatory drive may arise from noisy background processes rather than from specific sensory input. Analytical results may help to understand this more complicated situation of oscillatory noise and sensory stimuli being present at the same time. Put differently, before we can characterize the signal transmission of such a cell, it is in many cases beneficial to first thoroughly understand its spontaneous (i.e. autonomous signal-independent) activity caused by intrinsic noise or massive synaptic background. Thirdly, the temporal structure of single neuronal spike trains is conserved even if many independent spike trains are superposed [15] (weak correlations between neurons will additionally shape the power spectrum of the sum). Hence, on the network level, characteristics like the ISI density and ISI correlations of presynaptic cells driven by stochastic oscillations still affect postsynaptic target cells and thus shape network dynamics. Last, by comparing the ISI statistics of real neurons to analytical expectations, it may in certain cases be possible to draw conclusions about intrinsic parameters of the neural dynamics, which may otherwise be inaccessible, as has been carried out recently for sensory neurons with spike-frequency adaptation [16], [17].

Extensive experimental results pertinent to this problem of how stochastic oscillations shape the spontaneous spiking of a sensory neuron exist for the peripheral electroreceptors in paddlefish, which embed two distinct types of stochastic oscillators, one running at approx. 25 Hz, residing in a population of epithelial cells, which drives another in the peripheral terminals of afferents, running at approximately twice higher frequency. It was shown that the forcing from stochastic epithelial oscillations leads to rather complicated firing statistics of afferent firing, with multiple peaks in spike train power spectra, and extended-range correlations in the ISI sequence, continuing for tens of ISIs [18], [19]. We made use of a database of digitized recordings of spontaneous firing of electroreceptor afferents, obtained from *in vivo* paddlefish preparations in which external environmental noise due to water motion was minimized.

In this paper, we present novel analytical results for the firing statistics of a perfect integrate-and-fire (PIF) neuron model, which is driven by noisy oscillations [14]. The PIF model is the canonical model for a supra-threshold, regularly firing neuron, in which the effective mean input current 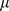 is so strong that it overshadows any voltage-dependence of the subthreshold membrane dynamics. The membrane potential 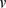 obeys the dynamical equation
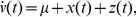
(1)where 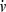 denotes the temporal derivative of 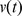. The model generates spikes whenever 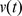 hits the threshold at 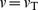 and is subsequently reset to 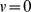. The driving consists of a so-called harmonic noise, representing the stochastic oscillation, given by the following Langevin equations [20]

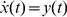
(2)

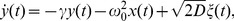
(3)together with a short-correlated Ornstein-Uhlenbeck process [21]

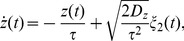
(4)which mimics broadband intrinsic fluctuations. The values of the noise are *not* reset after spiking. Important parameters of the model are: (i) the frequency ratio 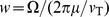 of the damped frequency 
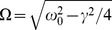
 of the harmonic noise to the mean firing rate 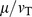, (ii) the quality factor 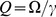 which quantifies the bandwidth and coherence of the harmonic noise, (iii) the non-dimensionalized variance of the harmonic noise 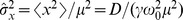, (iv) the non-dimensionalized variance of an Ornstein-Uhlenbeck (OU) broadband noise process 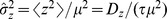, and (v) its non-dimensional correlation time 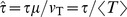. Our model with stochastic oscillations is illustrated in Fig. 1. Note that it can be regarded as a generalization of previous models, in which a PIF model was driven by uncorrelated white noise [22], exponentially correlated noise [16], [23], or a white noise and periodic driving [8], [12], [24], [25].

For this simple model, we calculate approximations for the ISI density and the ISI serial correlations and compare them to numerical simulations of the model. When discussing our explicit results, we focus on changes of the ISI statistics upon varying the ratio 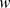 of the frequency of stochastic oscillations to the neuron's firing rate, a parameter that also shows a remarkable effect for the electroreceptor afferents of paddlefish. In particular, we show that upon variation of 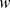 the skewness of the ISI density and also the structure of the ISI correlation coefficient as a function of the lag both change drastically, changes that are well-described by our theory.

We then compare our formulas for the ISI statistics to experimental data from electroreceptor afferents of paddlefish, obtained previously [18]. The analytical results from our simple perfect integrate-and-fire model work reasonably well in predicting (matching) these experimental data, indicating that the limitations of this model are not severe for representing sensory neurons with a high ongoing firing rate. This accords with other reports of remarkably good performance of stochastic perfect integrate-and-fire models for mimicking the ISI statistics of spontaneously active sensory neurons [17], [22] (for the performance of more general IF models in reproducing spike statistics, see e.g. [26], [27]). We conclude with a short discussion of the implications of our results for oscillatory physiological systems in general.

## Results

In this work, we aim at (i) the statistics of individual interspike intervals (ISI) by means of their probability density function (pdf), its coefficient of variation (CV), and its skewness, and (ii) the correlations between ISIs as quantified by the serial correlation coefficient (SCC). We study these statistics for the perfect integrate-and-fire (PIF) model and compare the theoretical results to experimental data.

### Perfect integrate-and-fire model

Despite the apparent simplicity of the PIF model, the fire-and-reset condition severely complicates the analysis. For the calculation of the ISI density and ISI correlations, one has to solve a first-passage-time problem in the form of a Fokker-Planck equation in a four-dimensional state-space spanned by the voltage and all the noise variables. The fire-and-reset condition imposes a complicated boundary condition on a half-space [28], which however can be ignored in the case of a weak colored noise where the standard deviation of the total noise is much smaller than the base current 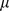, or, in terms of a small parameter 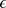, if
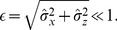
(5)In this case, based on the methods presented in [16], [23], the solution with natural boundary conditions can be used to calculate the ISI density. Furthermore, to obtain explicit expressions for the ISI moments and the SCC, a perturbation calculation of the characteristic function, in which 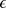 enters as the small parameter, turns out to be advantageous. These approximations are outlined in Methods and lead, for the considered problem, to formulas of reasonable length for the statistics of interest. In the next section, we compare our formulas to results from numerical simulations for small fixed values of 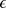. In Methods, we also show some of the statistics as functions of our small parameter 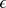 in order to give the reader some intuition about the range of validity of our formulas.

Our results are valid for arbitrary time scales of harmonic noise and OU noise; the general formulas are provided in the Methods section. However, because the effects of an exponentially correlated noise on ISI statistics are well-known [16], [23], [29], we focus on variations in the time scales of the harmonic noise, and set the correlation time of the OU noise to a small value if not stated otherwise. In most of the cases discussed, the latter noise thus acts essentially as a white-noise source. Direct inclusion of a white noise is not possible in our perturbation approach.

#### Shape of interspike interval density; skewness and coefficient of variation

For the ISI density with weak total noise, a short-correlated Ornstein-Uhlenbeck process 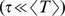, and a high quality factor of the harmonic noise 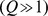, we obtain (see Methods):
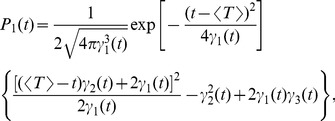
(6)with
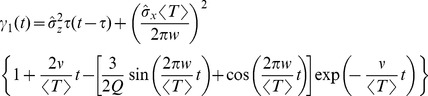
(7)

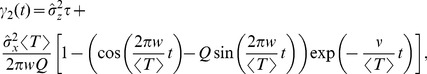
(8)

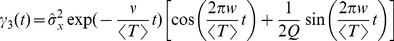
(9)

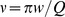
(10)where 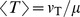 is the mean ISI. A more lengthy expression that holds true for arbitrary correlation time of the OU noise and arbitrary quality factor of the harmonic noise but still requires that the total noise is weak, is given in the Methods section in Eqs. (43–46).

**Figure 2:**
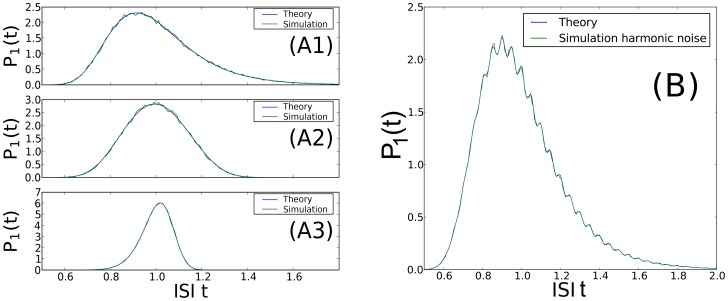
Comparison of ISI distributions 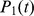 obtained from numerical simulation or theory Eqs.(6–9) for A and Eqs. (43–46) for B. **A**: ISI distributions for different frequency ratios: 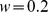 (top panel), 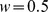 (middle panel), or 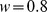 (bottom panel). Parameters: 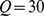, 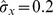, 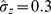, and 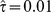. **B**: Example of a multimodal ISI histogram at high relative driving frequency 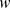, with harmonic noise input that was nearly periodic (large quality factor 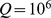). Parameters: 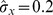, 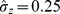, 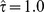, 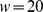, 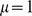, 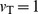 and, consequently, 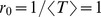.

**Figure 3:**
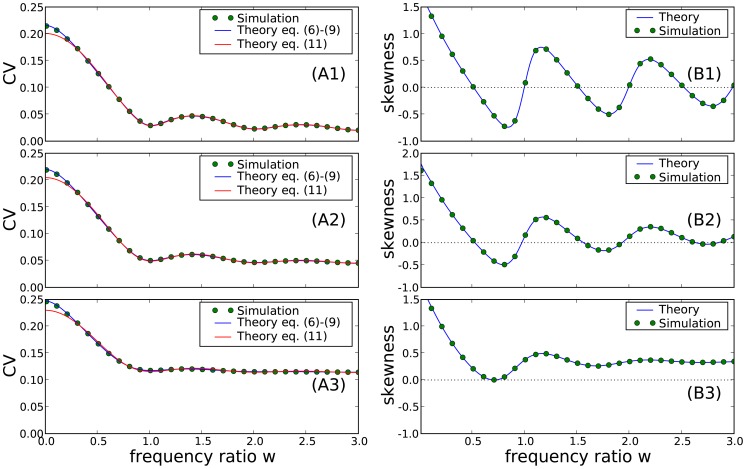
Second and third-order interval statistics as a function of the frequency ratio for different values of the OU broadband input (remaining parameters 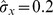, 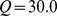, and 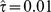). **A**: Coefficient of variation (CV) for 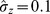 (A1), 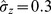 (A2), or 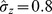 (A3). **B**: Skewness 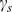 for 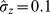 (B1) 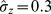 (B2), or 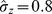 (B3). Theoretical CV and skewness (blue) were computed by numerical integration from the theoretical ISI distribution Eq.(6)–Eq.(9) ; the simpler expression Eq.(11) is shown in red in **A**.

Fig. 2A shows how the skewness of ISI distributions changes for different values of the frequency ratio 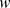. These examples suggest that the ISI distribution is positively skewed for 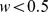, symmetrical for 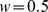, and negatively skewed for 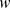 slightly larger than 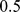. In fact, Fig. 3B reveals an oscillating pattern of the skewness 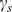 as a function of the frequency ratio. For sufficiently weak OU noise, the skewness is negative if 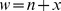 with integer 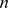 and 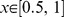, whereas the skewness is positive if 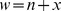 with integer 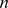 and 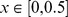. For stronger OU noise, the skewness is increased such that it is positive for all values of 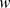 (cf. Fig. 3B3), as shown in Results for the electroreceptor afferents. This is plausible because it is known that with dominating exponentially correlated noise or with uncorrelated noise, the ISI density is positively skewed [16].

Note that the ISI distribution can also be multimodal, as demonstrated in Fig. 2B (here, the full theory Eqs. (43–46) had to be used, because 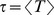, which is not small as assumed for Eq.(6)). Such multimodal histograms have also been obtained for the FitzHugh-Nagumo and leaky integrate-and-fire models driven by white noise and a strictly periodic signal, and have been experimentally observed for auditory neurons (see, e.g. [7], [30]) and electrosensory neurons in weakly electric fish (see, e.g. [31]).

Besides the skewness of the distribution, its relative width as quantified by the coefficient of variation (CV) is another important statistic, which characterizes the variability of ISIs. For a sufficiently high quality factor 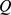 of the harmonic noise, and a small correlation time of the broadband OU noise, we obtain the following approximation for the squared CV:
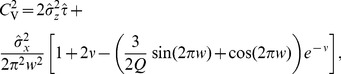
(11)an expression that yields values close to simulation data and to values of the CV obtained by using an integral involving our approximation for the ISI density Eq.(6) (cf. red lines to symbols and blue lines in Fig. 3 A).

We can draw a few conclusions from Eq.(11). Firstly, the OU and harmonic noise processes make independent contributions to the ISI variability, the two terms being proportional to 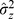 and 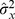. This is reasonable because it reflects the addition of the noise sources in the input, and the fact that we used a perturbation theory. Secondly, the CV is an oscillatory function of the frequency ratio 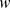. We can certainly expect that driving the system with a multiple of its own (firing) frequency allows for more regular spiking of the neuron (corresponding to the minima in the CV at 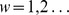) than a forcing with a frequency very different from its autonomous rhythm. Thirdly, there is an overall decline of CV with growing 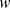 because of the exponential function. This is explained because the noise intensity of the driving stochastic oscillations declines with increasing frequency 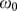, and hence the harmonic noise becomes less efficient in broadening the ISI density. All of these predictions of the formula are quantitatively confirmed for different values of the standard deviations of the OU noise (cf. Fig. 3A). Remarkably, for a perfect integrate-and-fire model, although the mean firing rate does not depend on the noise [23] (see also Methods section)and, in particular not on 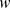, higher-order statistics such as the CV and the skewness do.

#### Serial correlation coefficients

The serial correlation coefficient (SCC) 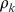 of interspike intervals can be computed from the variances 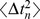 of the *n*th-order interval distribution (see Methods). For a high quality factor of the harmonic noise and a small correlation time of the OU broadband noise, the formula for the SCC can be considerably simplified to read
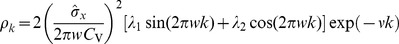
(12)with parameters 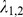 that do not depend on the interval lag 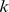:
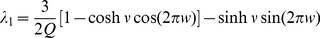
(13)

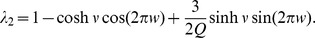
For the CV in Eq. (12), the approximate expression in Eq.(11) is adequate.

The dependence of the serial correlation coefficient Eq.(12) on the lag 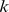 has the form of a damped oscillation, sampled at discrete values of the lag. This simple structure permits a number of conclusions. Firstly, if assumed to have a short correlation time, Ornstein-Uhlenbeck noise affects the SCC mainly via the CV in the prefactor, and thus tends to *reduce* the amplitude of the serial correlations at all lags. In contrast, an increase in the variance of the harmonic noise *amplifies* the serial correlations. Secondly, spike intervals are correlated mainly due to the correlations in the driving stochastic oscillations, and hence the SCC shows an overall decay with the “lag constant” 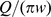, reflecting simply the finite (limited) phase coherence of the harmonic noise input. Thirdly, the SCC oscillates with the lag 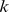, but because the argument 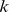 attains only integer values, rather complex looking patterns can result if the multiplying factor 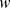 in the trigonometric functions is not an integer or a simple ratio such as 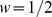. Fourthly, for a frequency ratio of 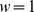, the SCC is close to zero for all lags because in this case the sine term in Eq.(12) drops out, and the prefactor of the cosine term 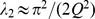 is rather small for a high coherence of the harmonic noise. Hence, if the frequencies of the stochastic oscillation and the neuron coincide, the resulting spike train is close to a renewal process. This is similar to findings in a bistable system under dichotomous driving [32], [33], for which the linear correlations vanish if the switching rate of the dichotomous driving coincides with the spontaneous hopping rate of the bistable system.

**Figure 4:**
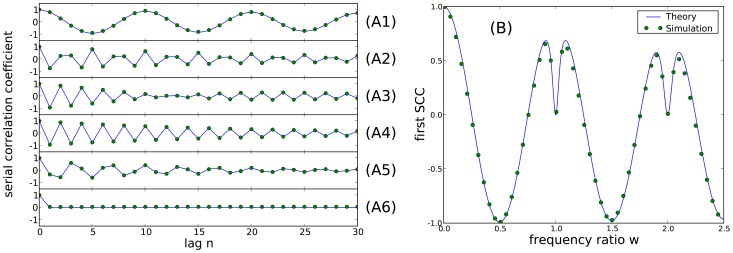
Serial correlation coefficients. **A**: SCC value (ranging from −1 to +1) as a function of the lag 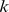 of interspike intervals, for different values of the frequency ratio: 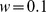 (A1), 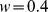 (A2), 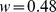 (A3), 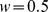 (A4), 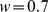 (A5), or 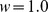 (A6). Parameters: 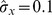, 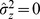, and 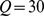. Dots: simulation. Lines: theory. **B**: SCC at lag 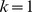, 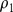, for highly coherent harmonic noise, as a function of 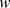. Parameters: 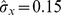, 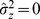, 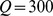. Theoretical curves were computed from Eq.(11) and Eq.(12).

All of these predictions are confirmed in the comparisons to numerical simulations in Fig. 4 A. Correlations are very small for 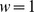 (Fig. 4A). They look complex for 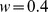 and 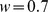, and are simply sinusoidal for small 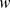. For a value 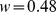, close to one half, a kind of beating pattern emerges. Finally, for 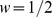, we observe a clear and long-extended oscillation of the SCC, alternating between positive and negative values, while decaying. In all cases, our formula works very well in predicting these different structures.

Another validation of our analytical result at very high coherence of the harmonic noise is illustrated in Fig. 4 B, showing the correlation coefficient of adjacent intervals 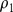 as a function of the frequency ratio. Also in this case, a nontrivial dependence is observed, with clear minima of the SCC at 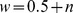 (where 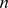 is an integer), and sharp changes around integer values of 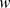.

**Figure 5:**
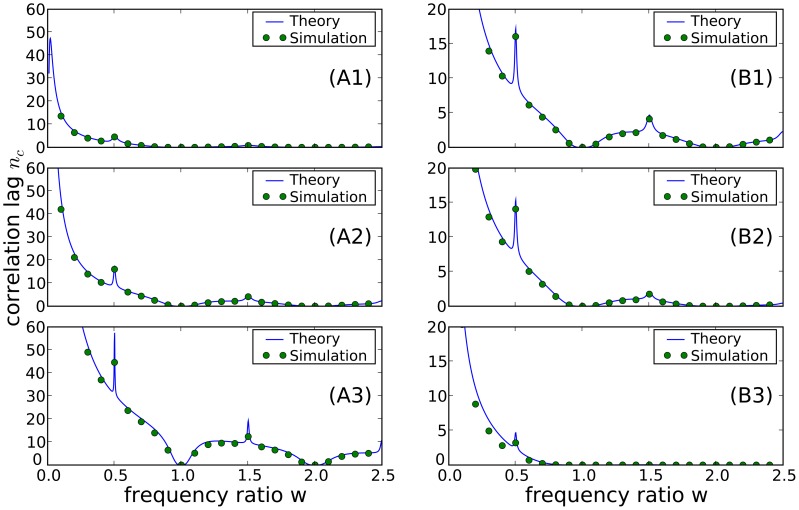
Correlation lag 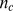, in units of the mean interspike interval, as a function of the frequency ratio 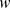. Dots show results of numerical simulations; blue lines show theory according to analytical evaluation of the sum Eq.(14). **A**: Correlation lag for different values of the quality factor of harmonic noise input: 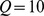 (A1), 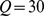 (A2), and 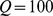 (A3). Parameters: 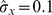, and 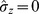. **B**: Correlation lag at different levels of OU broadband noise: 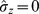 (B1), 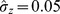 (B2), and 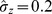 (B3). Parameters: 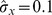, 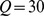, and 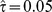.

The overall length of the ISI correlations can be characterized by the correlation lag, defined as
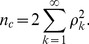
(14)The correlation lag measures the temporal extent of the SCCs, irrespective of the sign of the coefficients, in units of the mean interspike interval. For the SCCs approximated by Eq.(12), the sum in Eq.(14) is calculated exactly as:
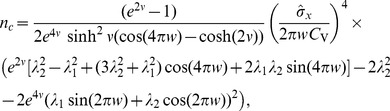
(15)where 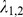 are given by Eq.(13). Fig. 5 shows the correlation lag versus the frequency ratio, 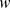. It is clearly maximal and even diverges for 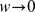. However, there are sharp local maxima around 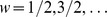. There are also minima close to zero for integer values of 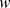 because in this latter case the spike train is nearly a renewal process.

### Comparison with experimental data from paddlefish electroreceptors

The theory developed in the previous sections was applied to experimental data obtained from *in vivo* electroreceptors of paddlefish. A single peripheral electroreceptor (ER) in paddlefish embeds two distinct oscillators. One resides in a population of epithelial cells (epithelial oscillator, EO), and can be recorded near an epithelium. This EO is coupled synaptically to another oscillator associated with the terminal of a given primary sensory afferent neuron (afferent oscillator, AO) [18]. Unidirectional coupling of these self-sustained oscillators, EO→AO, results in spontaneous biperiodic firing patterns of afferents having two fundamental frequencies, including the EO's at about 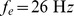, and another corresponding to the mean firing rate of an afferent, 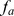, ranging from 30 up to 78 Hz, depending on the particular electroreceptor [18]. These two fundamental frequencies are seen as separate peaks centered at 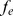 and 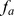 in the power spectral density of an afferent's firing. These peaks were used to determine the frequency ratio of the two oscillators as 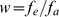. Only the AO is affected by external electric field stimuli. The EO acts as a stimulus-independent source of narrow-band noise input to the AO [34], [35]. Thus, the paddlefish electroreceptor system is an appropriate source of experimental data for validating the theory developed here.

In the *in vivo* preparation of paddlefish, an extracellular single unit recording offers information about the firing of an ER afferent. However, parameters of the epithelial oscillator, such as its effective quality factor and its variance, are hidden (Discussion). Previous computational studies have shown that a model of two unidirectionally coupled oscillators reproduces well the spontaneous and response dynamics of paddlefish ERs [14], [19], [36]. Here, we use our theory for the PIF model with harmonic noise, and in particular analytical expressions for the SCCs, to extract statistical and dynamical properties of the oscillators embedded in these ERs, and to verify the theoretical predictions of how the statistics of ISIs depend on the parameters of the coupled oscillators.

**Figure 6:**
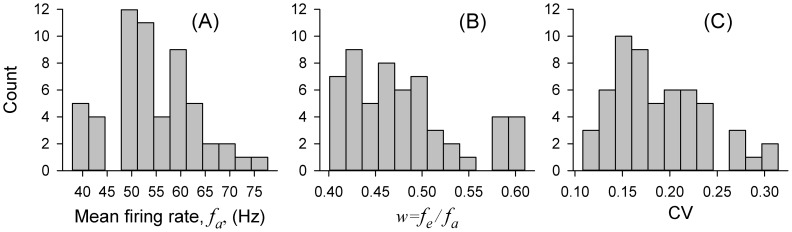
Histograms of firing statistics for a sample of 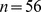 paddlefish ER afferents, including distributions of the mean firing rate, 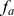, A, the ratio of EO to AO frequencies, 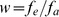, B, and the coefficient of variation, CV, C. These graphs are for different number of afferents than used in Fig. 6 of Ref. [18].

We analyzed spontaneous spiking activity from a sample of 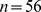 ER afferents (Methods). External noise was minimized, and a criterion for stationarity of long data segments was imposed. The data were in the form of sequences of spike times 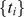, 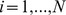, where 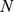 was of the order of 15000–50000 spikes, corresponding to recording times of 300–900 s. The mean firing rates of these units were in the range of 37.9–77.7 Hz, with mean and SD of 54.3±8.71 Hz. The ratio of the EO to AO frequencies, 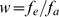, was 0.48 ± 0.06 for the sample (range 0.40–0.61). The CV of the corresponding ISIs of these units was 0.19 ± 0.05 (range 0.11–0.31). Histograms in Fig. 6 summarizing these statistics illustrate the diversity of firing rates and variability among this sample of ER afferents.

**Figure 7:**
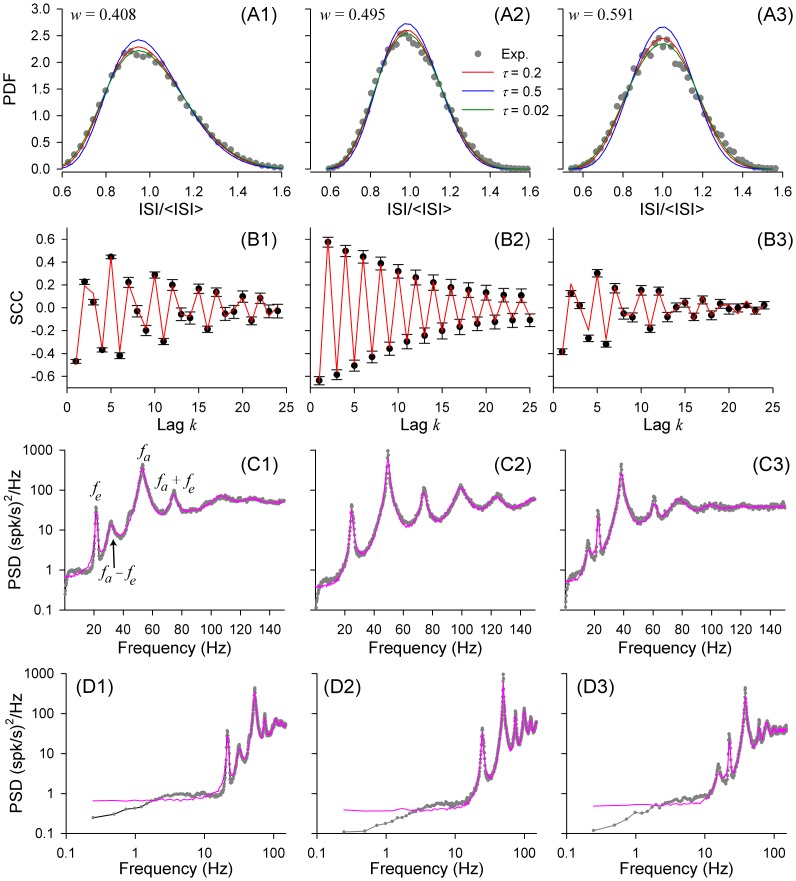
Experimental data from three representative paddlefish electroreceptor afferents (dots in A1–A3, dots and error bars in B1–B3, and gray lines in C1–C3), compared to theory, for the values of the frequency ratio, 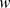, listed at the top. **A**: ISI probability density functions (PDFs). Theoretical PDFs (red, blue, green lines) were calculated using Eq.(6) with 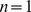; 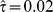, 0.2 and 0.5 (legend in A2), and other parameters derived from fitting the SCCs, as explained in the Methods, final section. **B**: Serial correlation coefficients (SCCs). Theoretical red lines show least square fits using Eq.(12). **C–D**: Power spectral densities (PSDs). Theoretical lines (magenta) were obtained from numerical simulation of the PIF model using Eqs.(1–4), with 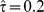, and other parameters the same as for theoretical curves in panels A1–3, derived from the SCC fitting procedure.

From a given spike sequence, we estimated the SCCs, the probability density of ISIs, and the power spectral density of the spike train. Fig. 7 shows these measures for three representative afferents with distinct values of the EO-to-AO frequency ratio, 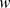, which were below, near, or above 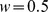. For all afferents in the sample, the distributions of ISIs were unimodal, and peaked close to the mean ISI (Fig. 7A). They all showed extended decaying series of significantly non-zero serial correlations, arising from the interaction of the EO and AO, with a structure determined by the frequency ratio 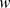 [19]. To assess the variation of SCC values due to unavoidable minor non-stationarity, we split the spike train into 20 segments, each 2000 ISIs long, and estimated the SCCs for each segment, which yielded error bars for the SCC values shown in Fig. 7B. The PSD (Fig. 7C) showed a characteristic structure of peaks, with a peak at the fundamental frequencies of the EO and AO (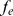 and 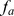, respectively), sideband peaks at combination frequencies (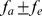), and their higher harmonics [18], [19].

To apply our theory to experimental data, we extracted from the serial correlations of an afferent spike train the four parameters needed for the PIF model: the quality factor 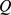 of the EO (a metric of the bandwidth and coherence of harmonic noise), the frequency ratio 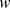, the SD of the EO 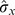 (the magnitude of harmonic noise), and the intensity of broadband OU noise, 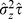. These parameters were extracted using a fitting procedure described in the Methods (last section).

Fig. 7 illustrates the outcome for three representative ER afferents. Our theoretical expression for the serial correlations of ISIs, Eq.(12), shown by the red lines in Fig. 7B, provided excellent fits for experimental data, as fitted values were within the error bars of most experimental SCCs. The extracted parameters of the PIF model for these three afferents are listed in Table 1.

**Table 1:** Fitted values of PIF parameters for the three afferent neurons for which ISI statistics are shown in Fig. 7.

Afferent	Mean firing rate (Hz)	CV	*w*	*Q*	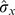	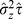
1	53.00	0.181	0.408	16.40	0.197	5.10×10^−3^
2	49.42	0.153	0.495	22.38	0.198	3.20×10^−3^
3	38.29	0.164	0.591	19.38	0.224	6.10×10^−3^

To calculate the probability densities of ISIs and the PSDs, we needed to accept a value for the correlation time of OU broadband noise, 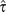 (which was 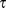 in units of the mean ISI interval, i.e. 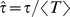). This was the only free parameter in our procedure of comparison of experimental data and theory. Probability densities of ISIs calculated according to the theory Eq.(6) (solid lines in Fig. 7A), with the parameters from Table 1, showed good correspondence with experimental data, and weak dependence on the correlation time of OU noise. Instead of tuning up 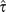, the correlation time of OU broadband noise was assumed to be fixed at 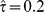 for all afferents, which provided good correspondence of experimental and theoretical ISIs distributions for all units, such as those shown in Fig. 7A.

Finally, Fig. 7C,D compares power spectra of spike trains obtained from numerical simulations of the PIF model Eqs.(1–4), using parameters from Table 1, to the PSDs of ER spike trains. Although the PSD's from simulations reproduced well the overall shape of experimental PSDs, the agreement between them is incomplete, especially at low frequencies, 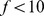 Hz, suggesting that the PIF model is an oversimplification of the stochastic dynamics of these electroreceptors. In particular, ER afferents in another fish species are known to exhibit spike-frequency adaptation resulting in short-term negative correlations [37]–[39]. These anticorrelations result in reduced power at low frequencies and a sharper peak at the mean firing rate. A previous study [19] showed that introduction of spike-frequency adaptation in a spiking model of paddlefish ERs results in an additional subtraction of low-frequency power similar to that observed in the experimental PSDs shown in double log scale in Fig. 7D. Nevertheless, the overall agreement of our simple and analytically tractable model is clear.

**Figure 8:**
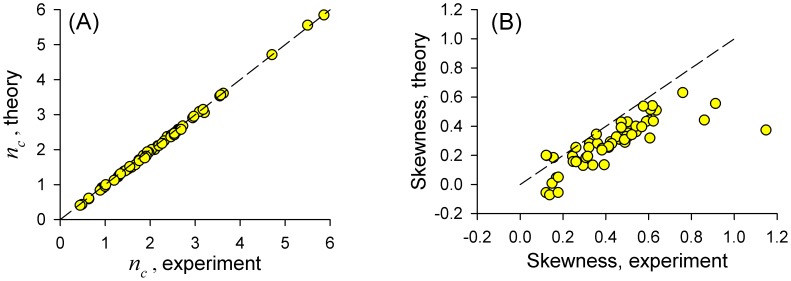
Comparison of experimental data with theory for the sample of 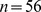 paddlefish ERs. **A**: ISI correlation lags 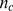 calculated from the experimental data according to Eq.(14), vs. values from theory, Eq.(15), calculated using parameters obtained from fitting experimental SCCs. **B**: Skewness of experimental vs. theoretical ISI distributions. 45° line is shown by dashed strokes on both panels.

The quality of fit is further illustrated in Fig. 8A showing measured and calculated correlation lag of the ISI sequence. Furthermore, correspondence of theory and experiment is demonstrated in Fig. 8B for the skewness of the ISI distribution, an independent variability measure derived from ISI distribution. As seen from the figure, theory estimate was biased towards somewhat smaller values of the skewness. The Spearman rank correlation coefficient was 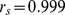 for the correlation lags and 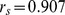 for the skewness (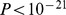 for both).

The application of the fitting procedure to our sample of ERs provided the following sample-averaged values for the parameters of the PIF model:


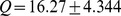
 (range 8.570–29.46), 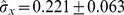 (range 0.129–0.443), and 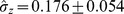 (range 0.084–0.303). It is noteworthy that the SD values of the broadband OU noise, 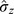, were close to or even larger than SDs of the harmonic noise, 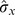. Nevertheless, the first-order approximation used in our theory was adequate to provide close correspondence with the experimental data, as seen in Figs. 7 and 8.

**Figure 9:**
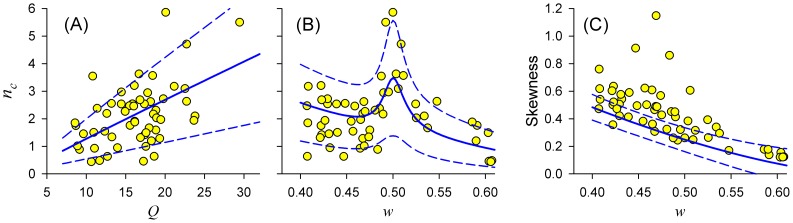
Statistical properties of afferent ISIs (ordinates) versus parameters of epithelial oscillations (abscissas), estimated from experimental data for the sample of n = 56 paddlefish ERs (filled circles). **A,B**: The ISI correlation lag characteristic, 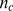, versus values of 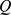 (A), or the frequency ratio 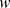 (B), of epithelial oscillations. **C**: Skewness of ISI distributions versus frequency ratio 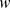. Blue lines: Theoretical results from PIF models for parameters extracted from each ER by fitting (Methods, final section), while varying 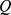 or 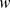. Solid blue lines: Mean curves for the sample. Dashed blue lines: 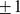 standard deviation.

Next, we analyzed how the statistical properties of afferent ISIs depend on the parameters of epithelial oscillations. In contrast to analytical or numerical analyses, which allow studying the dependence of a given statistical measure versus a single control parameter, other parameters being fixed, here instead each experimental data point on the scatter plots of Fig. 9 carries a set of 4 measurable parameters (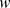, 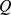, 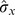, 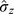) with fixed values. Variation of these parameter values between different ERs allows qualitative tendencies to be clearly seen in the sample of 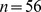 experimental data points, and these trends can be compared to theoretical predictions. We start with Fig. 9A, showing a scatter plot of the electroreceptor ISI correlation lag 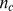 (i.e. how slowly the serial correlations of ISIs decayed; Eq.(14)) versus the 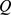 values of epithelial oscillations (i.e. their bandwidth and coherence). According to the theory, SCCs will decay more slowly for more coherent epithelial oscillations (i.e. for larger 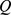 values), such that 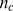 increases with 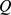. This prediction was supported by a positive correlation between them in the experimental data (Fig. 9A), having a significant Spearman rank correlation coefficient of 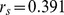 (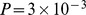), despite considerable scatter.

To quantitatively compare experimental data to expectations from theory, we calculated 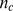 versus 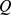 from the PIF model Eq.(15) for each ER in the sample, with the other three parameters (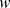, 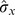, 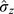) extracted using the SCC fitting procedure (Methods, final section), yielding a curve for that electroreceptor over the range of 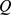 values along the abscissa of Fig. 9A. The family of n = 56 PIF model curves were then averaged to calculate the sample-averaged tendency 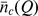 (solid blue line) and its standard deviation 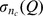 (dashed blue lines):
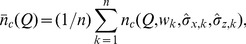
(16)

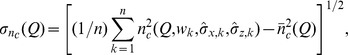
where 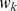, 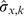, and 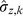 are the parameters for the *k*-th afferent. The mean trend from theory formed a straight line with positive slope, correctly predicting that the correlation lag increases for more coherent epithelial oscillations. More than expected of the experimental data points (47/56 = 84%) fell within the predicted ±1 SD (68%) bands.

The ISI variability metric 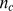 also depended strongly on the frequency ratio 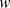 (Fig. 9B), with the largest correlation lag attained for a value of 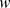 close to 0.5, i.e. when there were two afferent spikes per cycle of epithelial oscillations. This is consistent with the PIF theory (solid blue line) showing a local maximum of the correlation lag for 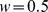. Furthermore, most experimental points were within theoretical ±1 SD bounds (blue dashed lines, calculated in the same way as for Fig. 9A but by varying 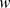 instead of 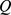). The large scatter of data points presumably was due to diversity of afferent variabilities.

Finally, Fig. 9C shows that the skewness of ISI distributions was negatively correlated with the frequency ratio 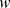, such that the tails of ISI distributions were significantly reduced at higher values of 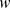 (Spearman correlation coefficient 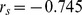, 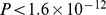). This negative correlation was borne out by analytical calculations from the PIF model (blue lines).

## Discussion

This report analyzed a scenario in which the membrane potential and spiking of a neuron is forced by weak noisy oscillatory input, in a narrow but non-vanishing frequency band. Our goal was to study the effects of narrow-band noise input on the output spiking statistics of a neuron. Our analysis centered around a perfect integrate-and-fire model of a single neuron, stimulated by a mixture of stochastic oscillations and broadband noise. We obtained novel explicit expressions for the probability density and serial correlation coefficients of the model's interspike intervals (ISIs). By a perturbation calculation of the Fokker-Planck equation, we derived a structurally simple form for the serial correlation coefficient. This novel derivation helps to solve the inverse problem: using the spike statistics of a neuron to estimate parameters of the underlying stochastic processes that drive its firing. No other body of theory has rigorously addressed the implications of narrow-band stochastic input for neural firing statistics, despite much acclaim of the widespread roles of oscillators in nervous systems.

Our new analytical formulas compare extremely well with results from our numerical simulations of the spiking neuron model, provided that the variance of the total input noise is weak, such that the coefficient of variation of spiking output remains low, less than approximately 0.3.

We compared the PIF theory to spike time data from a well-defined experimental system, the electroreceptor afferents of paddlefish, which receive stochastic synaptic driving in a narrow frequency band from ongoing oscillations arising in their sensory epithelia. For a given afferent's sequence of ISIs, a fitting procedure was used to extract four parameters needed for the PIF model, and the model's output was computed. The only appreciable discrepancy between model and experiment was observed in the skewness of ISI distributions in which the model showed consistently smaller values and in the low frequency regime of spike train power spectra, in which the model showed excess noise power. This low frequency regime is presumably shaped by spike-frequency adaptation, which we did not incorporate in our model to keep it analytically tractable.

The fitting parameters varied considerably for different units, reflecting natural variability of the electroreceptors. These natural ranges of values permitted us to check whether different functional relationships were correctly predicted by the theory. For example, in both theory and experiment, the temporal extent of the SCCs (i.e., their correlation lag, 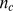) increases monotonically with the quality factor of epithelial oscillations (Fig. 9 A), whereas 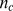 depends in a non-monotonic fashion on the frequency ratio 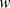, attaining a maximum at 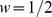 (Fig. 9 B), for both theory and experiment. We note that previous computational work showed that this frequency ratio corresponds to a maximum mutual information rate for electroreceptor afferents stimulated by a time-varying stimulus [36]. Thus, our study provides further arguments in favor of the idea that oscillators embedded in the electroreceptor system are tuned to maximize stimulus encoding [34].

We applied our formulas to the inverse problem of whether the spike statistics of a neuron can be used to estimate parameters of the underlying stochastic processes that drive its firing. Using only our formulas for the firing rate, the CV, and the serial correlation coefficient, we were able to predict the parameters of the epithelial oscillator (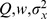) and the variance of the broadband noise, 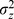. Using these predicted parameters, our analytical formulas provided excellent fits to the experimental serial correlation coefficients, and close correspondence between model and experiment in their ISI distributions and power spectra (except at low frequency).

It could be argued that the suggested solution of the inverse problem is too cumbersome in the case of epithelial oscillations of paddlefish ERs. Power spectra of afferent spike trains show a second fundamental peak due to synaptic input at the frequency of epithelial oscillations, 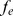, so this spectral peak provides direct information. However, the 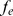 peak is of limited usefulness for measuring parameters of the epithelial oscillation such as their quality factor, 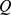, because afferent spike train spectra incorporate the effects of nonlinear transformations during synaptic transmission and spike generation, and also because the 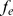 peak may overlap with other spectral peaks, e.g. a sideband.

In general, the good agreement of the simple PIF model and the experimental data indicates that the detailed voltage dependence of the neural dynamics, more faithfully modeled in a conductance-based Hodgkin-Huxley model, is less important for the spiking statistics than the stochastic oscillatory driving, provided that the mean input to the neuron is appropriate for tonic firing with low ISI variability. The PIF model is able to reproduce several complex features observed experimentally in the afferent spike timing [18], [19], including skewing of ISI probability densities in different ways, oscillations, beating, or seemingly chaotic patterns in the serial correlations of interspike intervals as a function of the lag. The ability of our theory to reproduce complex non-renewal spike timing may encourage experimentalists to look for and analyze seemingly complex looking patterns in ISI correlations.

Examples of narrowband noisy neural oscillations include the gamma band (25–90 Hz) extracellular field potentials prevalent in mammalian cortex [40], which have been suggested, along with transient synchronization between brain areas, to mediate or reflect higher cognitive functions [4], [41]. Such fast gamma oscillations interact with slower rhythms, including the theta rhythm in hippocampus, and slower oscillations in thalamic nuclei. From the point of view of a single cell in a specific brain region engaged in a specific rhythm, input from other brain regions could be regarded as a stochastic oscillation. What matters the most for the ISI statistics of this cell may be not so much the synchrony of the activity but the frequency ratio between the stochastic oscillatory driving and the mean firing rate of the driven cell. Put differently, instead of coherence and synchronization, an important signal for cognition might be the frequency ratio of narrowband stochastic oscillations in related brain areas. Our work provides a rigorous demonstration and model of how the operation and spiking statistics of neurons can change sharply when the frequencies of different stochastic oscillatory components approach or assume an integer ratio (i.e. a rational number). Perhaps integer ratioing could function as a trigger or gate for cognitive, memory, or other information processes, acting like an event detector.

Specifically, our results show how the structure of a neuron's serial ISI correlations depends characteristically on the frequency ratio of weak stochastic oscillatory input, and the intrinsic periodicity of a neuron receiving the input, with extreme SCC behavior occurring at integer multiples. We have delineated other parameters which strongly affect SCCs including the quality factor of stochastic oscillatory drive (i.e. its bandwidth and coherence), the neuron's mean firing rate, and the overall level of spike timing noise (its CV). Our results bear general importance for the effects of weak stochastic oscillations on the spiking statistics of neurons in other systems, and are relevant to the study of neuronal firing in many brain regions. We have defined a basis in theory for using serial correlations to detect and characterize weak interactions of physiological oscillators, which may apply to other organ systems as well [42]–[44]. For example, the breathing and heartbeat rhythms can assume integer frequency ratios, and are known to be coupled [45].

## Methods

### Statistics of stationary sequences of interspike intervals

We used conventional metrics, summarized here for clarity, to characterize the statistics of a stationary spike train given by the set of spike times 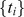. The spiking statistics can be derived from its sequence of interspike intervals (ISIs) 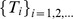, where 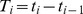 denotes the *i*-th ISI. Calculations are simplified without loss of generality by restricting the stationary ensemble of spike trains to those realizations having a spike at time 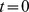, called the zero-th spike. Under this choice of the origin, the *n*-th order interval, defined as the sum of 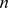 consecutive ISIs, is equal to the *n*-th spike time:
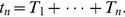
(17)The stationary spiking statistics can be formulated in terms of the statistics of the *n*th-order intervals, for all 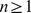. Knowing the probability density of the *n*-th-order interval
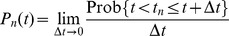
(18)for arbitrary 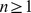, yields complete information about the spiking statistics.

The *ISI probability density* 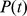 is given by the first-order interval density: 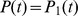. Let the mean ISI be denoted by 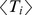, which is independent of the index 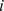 due to stationarity (here and in the following, the notation 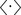 refers to the ensemble average). Then, the *mean* of the *n*th-order interval is 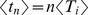, and the *variance* is 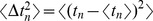. The *coefficient of variation* (CV), defined as the ratio between ISI standard deviation and mean is given by
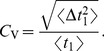
(19)The CV is a measure of irregularity of the spike train; it is equal to one for a Poisson process. The statistics of individual ISIs are further characterized by the *skewness* defined by
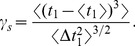
(20)Correlations among the ISIs are characterized by the *serial correlation coefficient* (SCC)
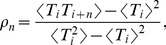
(21)which depends on the order of ISIs. The SCC measures the correlations between two ISIs that are lagged by an integer 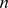. This measure can be related to the *n*th-order variances by the formula [23]

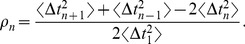
(22)


### Relation between noise parameters

In this paper we gave parameters of the model simulation in terms of 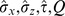 and 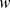. Here we provide the inverse relationship, how to obtain the simulation parameters 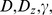 and 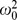 given 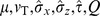 and 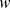:
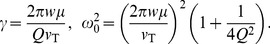
(23)Using these values, one can easily determine the noise intensities:
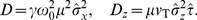
(24)


### Analytical formulas for a PIF model driven by weak stochastic oscillations

The mean ISI in the PIF model is independent of the properties of a noise with zero mean [23] and is given by
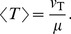
(25)In fact, for large times 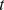, the spike count 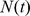 is determined by the free running solution of Eq. (1) (i.e. 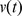 without resetting, cf. [29]): 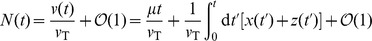. Averaging this expression, the integral term vanishes and we obtain the firing rate 
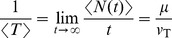
, from which follows Eq. (25). Furthermore, using Eq. (17), we find that the mean *n*-th-order interval is given by 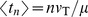.

To obtain higher moments as well as the probability density of 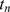, it is important to recognize that the *n*th-order intervals can be interpreted as a first-passage time (FPT). In fact, in the PIF model the statistics of the sum of 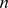 subsequent ISIs for a firing threshold 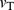 is equal to the statistics of a single ISI with respect to a firing threshold at 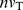. The statistics of a single ISI is, however, nothing else than the statistics of the FPT with respect to the boundary 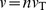 for a “particle” that starts at 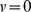 and is not reset at 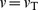. The equivalence between *n*-th spike time and the FPT with respect to the boundary 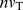 is due to the fact, that the “velocity” 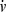 of the particle is independent of 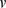 according to Eq.(1). Consequently, the time of the *n*th spike depends only on the total distance 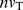 that a particle has to cover.

The FPT problem can be solved by using the Fokker-Planck equation for the probability density 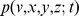, which is associated to our stochastic model (see e.g. [46]). This equation reads
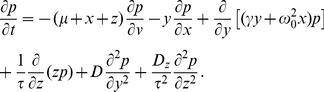
(26)The probability density has to satisfy certain boundary and initial conditions. Specifically, we demand that particles that have crossed the boundary 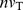 are not allowed to re-enter the domain 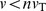 (see [28] for a discussion on a related problem). This precludes repeated threshold crossings. As a consequence, there is no probability flux *through* the boundary with negative velocity. Mathematically, this entails the boundary condition
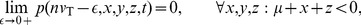
(27)because no particles are found just below the boundary 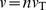 if 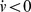. Furthermore, we require that the probability density and the probability current vanish at infinitely distant boundaries (natural boundary conditions). In the following, we assume that the total noise is weak. In particular, we require that the standard deviation of 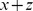 is much smaller than 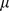, or
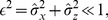
(28)where
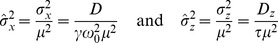
(29)are the normalized variances of 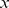 and 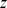. Under this assumption, it is highly unlikely that 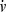 becomes negative and hence, the boundary condition Eq.(27) can be safely neglected.

The initial condition is determined by the fact that at time 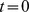 the neuron has just fired a spike and the membrane potential has just been reset to 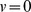. This implies, that the initial probability must satisfy
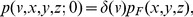
(30)where 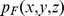 is the probability density of the variables 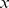, 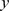 and 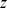, *upon firing*. How can one obtain this probability density? To this end, let us for the moment reconsider the original setup, where the trajectories are reset if 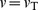. Then the dynamics are restricted to the domain 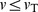 and the probability density 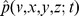 will in this case converge to some stationary probability density, which will be denoted by 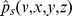. The density upon firing must be proportional to the fraction of particles that exit the domain through the surface element 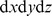 per unit time. This fraction is equal to 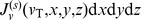, where 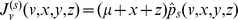 is the stationary probability current in the 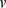 direction. Thus,
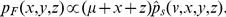
(31)Under the weak noise assumption Eq.(28), the stationary distribution 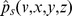 does not depend on 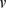, because for 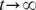 all values 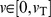 have equal probability due to the voltage-independence of the membrane dynamics and the loss of the memory about the initial condition. We hence find
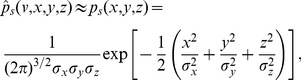
(32)where 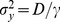. Upon normalization, the initial condition can now be written as
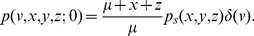
(33)


The time-dependent solution of the Fokker-Planck equation (26) with the initial condition (33) can be related to the *n*th-order interval density 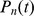 as follows: The probability per unit time to cross the boundary 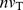 at time 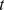 is equal to the total probability current across the boundary at time 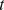, hence
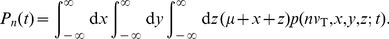
(34)For the sake of notational convenience, we will henceforth use the dimensionless time 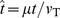 and membrane potential 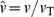. Furthermore, we introduce the non-dimensionalized variables
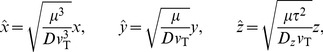
(35)and the non-dimensional parameters
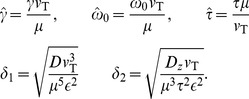
(36)In these rescaled variables the Fokker-Planck equation takes the form
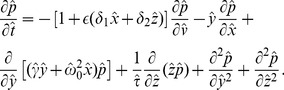
(37)


#### Probability density of the *n*th-order intervals

The *n*th-order interval density can be derived from the characteristic function
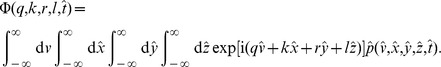
In fact, comparing with Eq.(34) we observe that 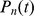 can be represented by the formula
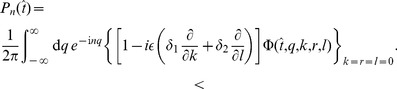
(38)Applying the respective Fourier transform to the FPE yields a first-order equation for the characteristic function 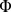
Applying the respective Fourier transform to the FPE yields a first-order equation for the characteristic function *Φ*:
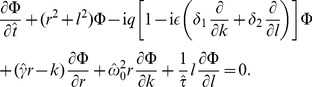
(39)The initial condition can be derived from Eq.(33) and reads
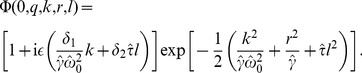
(40)To solve Eq.(39), it is useful to make the ansatz
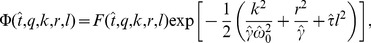
(41)which after insertion into Eq.(39) yields
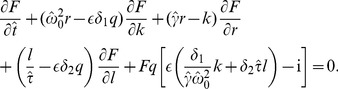
(42)This equation can be solved by the method of characteristics. Using Eq.(38), the final result reads (with 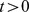)
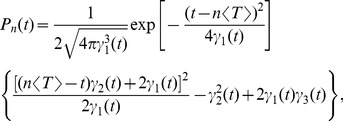
(43)where the functions 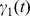, 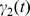 and 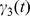 are given by
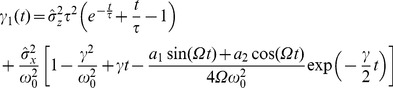
(44)

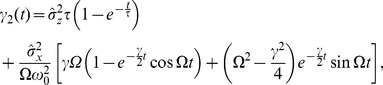
(45)

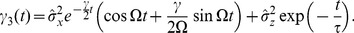
(46)and
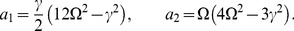
(47)


For stochastic oscillations with a high quality factor 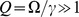, these expressions can be simplified. In this case, the damped and undamped oscillation frequencies are approximately the same, i.e. we can set 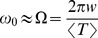. Assuming furthermore, that the correlation time of the OUP is much smaller than the mean ISI, i.e. if 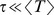, we can neglect the exponentials 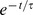, resulting finally in Eqs. (6–9).

#### Moments of the *n*th-order interval

To compute the *n*th-order variance, we consider the Laplace transform of the *n*th-order interval density
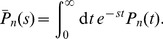
(48)Knowing this function the moments can be generated by
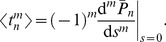
(49)The Laplace transform can be derived from the function
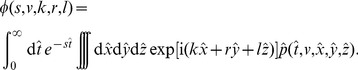
(50)In fact, from Eq.(34) we find that
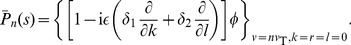
(51)


Applying the transformation (50) to the FPE (37) leads to an equation for 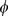:
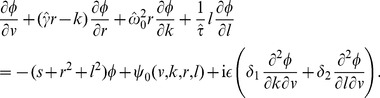
(52)Here, the function 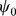 arises from the initial condition Eq.(33) and is given by
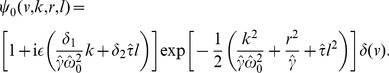
(53)
Eq.(52) is difficult to solve, because of the mixed derivatives on the right-hand-side. For weak noise, however, 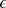 is a small parameter and perturbation theory can be applied. To this end, 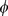 is written as a power series in 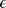, i.e. 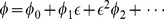. Substituting this expansion into Eq.(52) and solving order by order, yields an approximation of 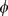 for weak noise. Using further Eq.(49), we obtain in the leading order the *n*th-order variance
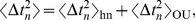
(54)where
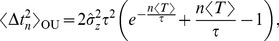
(55)

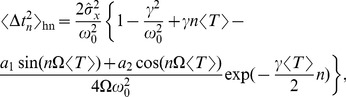
(56)For 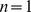, we find the squared CV in leading order of 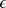 (cf. Eq.(19)):
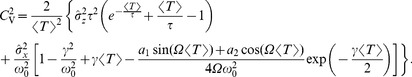
(57)Again, for a high quality factor 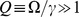, one can set 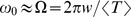, which yields
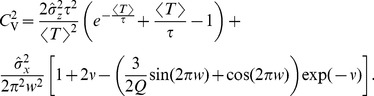
(58)Furthermore, if 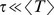 the term 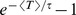 can be neglected in the first term of Eq.(58) leading to the simplified formula Eq.(11).

#### Serial correlation coefficient

Knowing the *n*th-order variance given above, the SCC can be computed using Eq.(22)

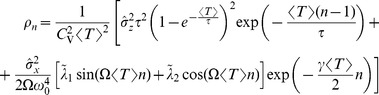
(59)with
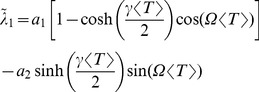
(60)

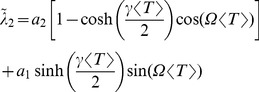
(61)and 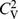 is given by Eq.(57). For small correlation time of the OUP, the first term can be neglected. Furthermore, if the quality factor of the harmonic noise is high, we can again use the approximation 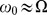. Under these assumptions, the SCC is given by Eq.(12) (see Results).

### Comparison of numerical simulations to theory for higher noise amplitudes

**Figure 10:**
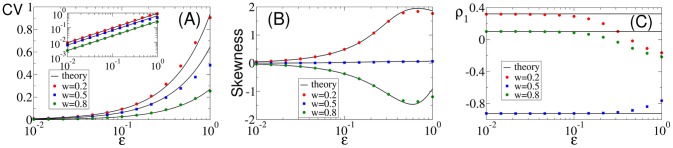
Comparison of ISI statistics from numerical simulation and theory versus noise strength 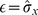 for different values of the frequency ratio 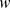 as indicated in the legends: coefficient of variation (A) with a double logarithmic plot of the same data in the inset, skewness of ISI density (B), and serial correlation coefficient at lag one (C). Remaining parameters: 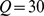, 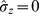, 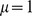, and 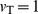.

Here we briefly discuss the range of validity for our approximations. In general, we expect our theory to be valid whenever 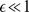. Let us recall that
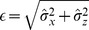
(62)and, hence, we can increase 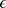 by increasing only 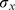, only 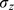, or both simultaneously. In Fig. 10, we chose the first option, i.e. we vary only the harmonic noise strength. In the three panels of Fig. 10, we show the CV, the skewness, and the serial correlation coefficient at lag one as functions of 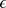 and for three selected values of the frequency ratio 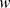. Varying both 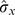 and 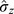 for the ratios 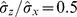 or 1 yield very similar results (not shown).

The plots illustrate that the theory works well for 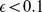, confirming its general validity. For the statistics of the single ISI (CV, skewness), only minor deviations are found even for 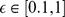. This is not so for 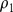, which shows strong deviations for 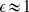 and can even reverse its sign for a strong harmonic driving at frequency ratios 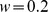 and 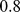. However, deviations of 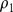 between theory and simulations can be neglected for 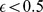 and 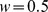, which covers the experimentally relevant ranges of 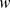 and 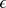 for paddlefish electroreceptor afferents (data for 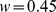 and 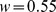 look very similar but are not shown).

### Data analysis for electroreceptor afferents

Data from 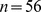 afferents of 19 animals were from experiments at University of Missouri-St. Louis in 2000–2002, under an IACUC-approved animal use protocol (W01-13) there. The spontaneous discharges of electroreceptor afferents of paddlefish (*Polyodon spathula*) were recorded in *in vivo* preparations with procedures detailed in [18]. A fish was held at rest in a plastic chamber, maintained by a stream of oxygenated water. The water temperature was maintained at 22°C. No external electric field or any other relevant kinds of stimulation were applied while recording spontaneous afferent firing. Disturbance of spontaneous afferent firing by the turbulence of water flowing into the mouth of a fish was minimized by partitioning the chamber [18]. Nonstationarity was further minimized by choosing segments of data in which a moving average of the afferent firing rate over a 10 s window fluctuated less than ±2% from the mean firing rate.

Analyses of spike time sequences from paddlefish electroreceptor afferents were performed using MATLAB's Signal Processing and Statistics Toolboxes. A spike train, 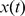, was represented as a sequence of delta functions centered at spike times of an afferent, with the mean firing rate 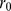 subtracted: 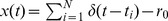. For the purpose of estimating the power spectral density (PSD), each delta function was approximated by a rectangular pulse of width 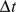 and height 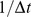, where the sampling interval 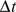 was set to 1 ms. The PSD, defined as 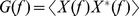, where 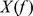 is the Fourier transform of the spike train, was estimated using the Welch periodogram method (function *pwelch* of MATLAB's Signal Processing Toolbox).

The following procedure was used to extract 4 parameters of the PIF model (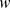, 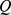, 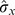, and 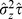) from an experimental sequence of ISIs:

- An original ISI sequence was normalized to have the mean ISI equal to 1, 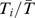 (where 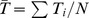 is the average of the sequence of 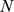 intervals)
- The experimental coefficient of variation and SCCs were calculated according to (19) and (21).
- The experimental SCCs were fitted using MATLAB function *nlinfit* with the formula (12), where 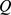, 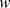, 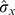 are fitting parameters. For the fitting, an initial value of the frequency ratio 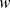 was estimated from the experimental power spectrum density as the ratio of the peak frequencies of epithelial to afferent oscillations, 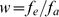, see e.g. Fig. 7(C1).
- Finally, the intensity of OU noise, 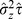, was calculated from Eq.(11), yielding
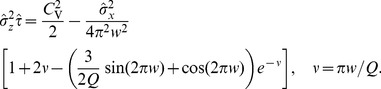
(63)
